# Effects of programmed flexor–extensor alternating electrical acupoint stimulation on upper limb motor functional reconstruction after stroke: study protocol for a double-blind, randomized controlled trial

**DOI:** 10.1186/s13063-023-07283-3

**Published:** 2023-05-11

**Authors:** Yang Liu, Xu Dong, Hong Huo, Liyuan Feng, Dan Tong, Jiahui Liu, Hongyan Zhang, Yingkang Zheng, Shuai Wang, Dongyan Wang

**Affiliations:** 1 Heilongjiang University of Chinese Medicine, No. 24 Heping Road, Xiangfang District, Harbin, People’s Republic of China; 2 The Second Affiliated Hospital of Heilongjiang University of Chinese Medicine, Nangang District, No. 105 AshiheRoad, Harbin, People’s Republic of China

**Keywords:** Stroke sequelae, Upper limb motor dysfunction, Programmed, Flexor–extensor, Acupoint, Electrical stimulation

## Abstract

**Background:**

Stroke’s prevalence and morbidity are increasing (Guano, et al. Neuro 89:53–61, 2017), and limb motor dysfunction is left in most patients (Gittler, et al. JAMA 319:820–821, 2018). Particularly, the rehabilitation of upper limbs is more difficult and time-consuming (Borges, et al. The Cochrane database of systematic reviews 10:CD011887, 2018).

**Methods:**

A double-blind randomized controlled trial (RCT) will be conducted to investigate whether a new functional electrical stimulation (FES) combined with acupoint therapy is more effective in the rehabilitation of upper limb motor dysfunction after stroke. Patients who meet the inclusion criteria will be randomly divided into two groups: programmed flexor–extensor alternating electrical acupoint stimulation group (PES group) and conventional flexor–extensor alternating electrical acupoint stimulation group (CES group), which will be treated for 3 weeks. The primary outcome measures are electroencephalogram (EEG) and surface electromyogram (sEMG). The secondary outcome variables include MBI (modified Barthel index), China Stroke Scale (CSS), FMA-U (Fugl-Meyer assessment upper limb), MMT (manual muscle testing), and Brunnstrom.

**Discussion:**

The results of this study are expected to verify the efficacy of PES therapy in the rehabilitation of upper limb motor function after stroke. This may promote the widespread use of the therapy in hospitals, communities, and homes for early and continuous treatment.

**Trial registration:**

ClinicalTrials.gov NCT05333497. Registered on April 11, 2022.

## Introduction

Global Burden of Disease (GBD) has indicated that stroke is the leading cause of death and disability among adults in China [1]. More than 80% of patients have suffered from acute motor dysfunction, and over 50% have long-term limitations [2]. Among them, about 69 to 80% of stroke patients have upper limb motor dysfunction [3]. During the recovery, the affected upper limb shows a flexion pattern and limited movement, which seriously influences the patient’s daily life. It is a key and difficult point of stroke sequela always, and a lot of exploration of different rehabilitation therapies and intervention effects has been conducted by domestic and foreign scholars. With the increasing recognition of traditional Chinese medicine (TCM), especially acupuncture, the therapies for stroke sequelae by integrated traditional Chinese and Western medicine have been widely used. In recent years, functional electrical stimulation (FES) has been a concern in the rehabilitation field. Therefore, the combination of FES and TCM acupoint therapy on upper limb functional reconstruction after stroke is worth further study.

FES is commonly used in patients with motor disorders such as stroke, spinal cord injury, and hemiplegia. The possible mechanism is mainly brain remodeling and activity-dependent plasticity [4]. By electrical stimulation, one or more groups of muscles are induced to generate muscle movement or mimic normal voluntary movement in order to improve the function of the stimulated muscles or muscle groups. Studies have shown that FES can help patients with neural dyskinesia rebuild some motor function [5] to achieve the purpose of rehabilitation, and can also help relieve muscle pain and spasm for the improvement of muscle status [6]. It has been widely used in the rehabilitation of hemiplegia patients and has very definite effects on the recovery of sensory and motor function of limbs.

Acupuncture is a vital part of TCM, as well as an internationally recognized physical therapy, with high safety, significant efficacy and no serious adverse events in limb motor rehabilitation after stroke [7]. Currently, the mechanism of an acupoint is not completely clear, but the viewpoint of central nervous control is an essence of the curative effect [8–11]. In this study, the flexor–extensor alternating electrical acupoint stimulation instrument has combined traditional acupuncture with modern low-frequency technology, with the advantage of non-invasion and convenience, to improve joint activity, relieve spasm, enhance muscle strength, and promote the central nervous system and limb function reconstruction [12]. However, simultaneous movement of several single joints is produced by local stimulation with the conventional instrument, but no coordinated movement of multiple joints is generated. In order to improve patients’ ability of daily living (ADL), the program is improved in this study based on the original instrument to a programmed one. Through the output of electrical stimulation at different times, muscles or muscle groups in different parts are stimulated to form programmed movement. By neuro-electrophysiological test and scale evaluation, this study will conduct a double-blind RCT to objectively evaluate the effect of flexor–extensor alternating electrical acupoint stimulation on upper limb motor functional reconstruction.

## Methods

### Pre-design

Five healthy subjects and five patients are selected to conduct a survey on the comfort of this instrument parameters, body position, stimulation time, muscle fatigue, completion of programmed movements, and other conditions and to refine the trial design in response to feedback and comments from participants.

### Study design

**Fig. 1:**
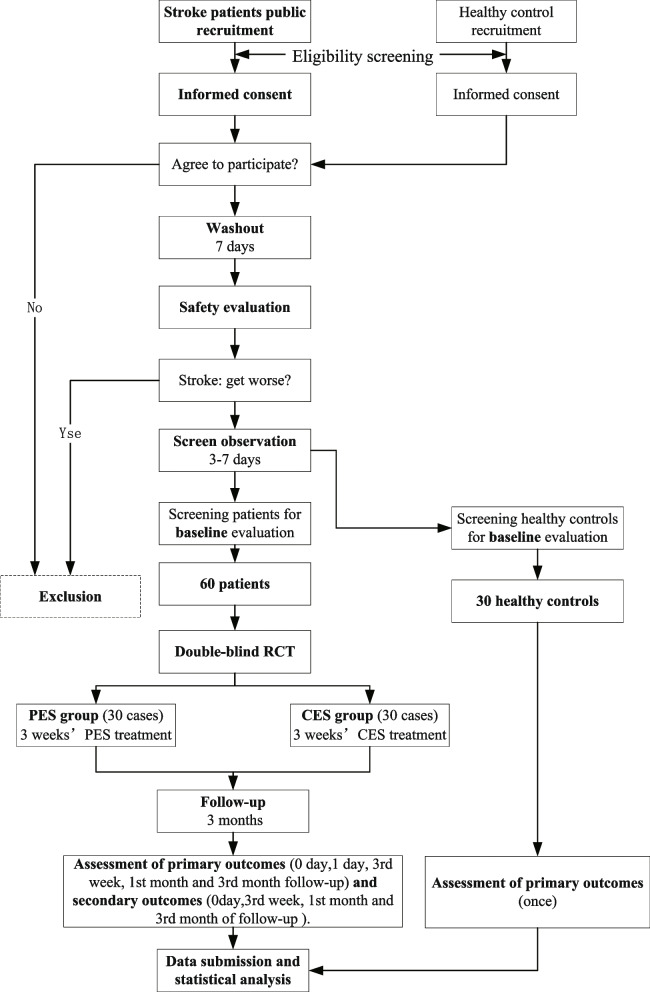
Flow diagram of the whole trial (details of patients’ and healthy controls’ groups in Figs. 2 and 3)

**Fig. 2:**
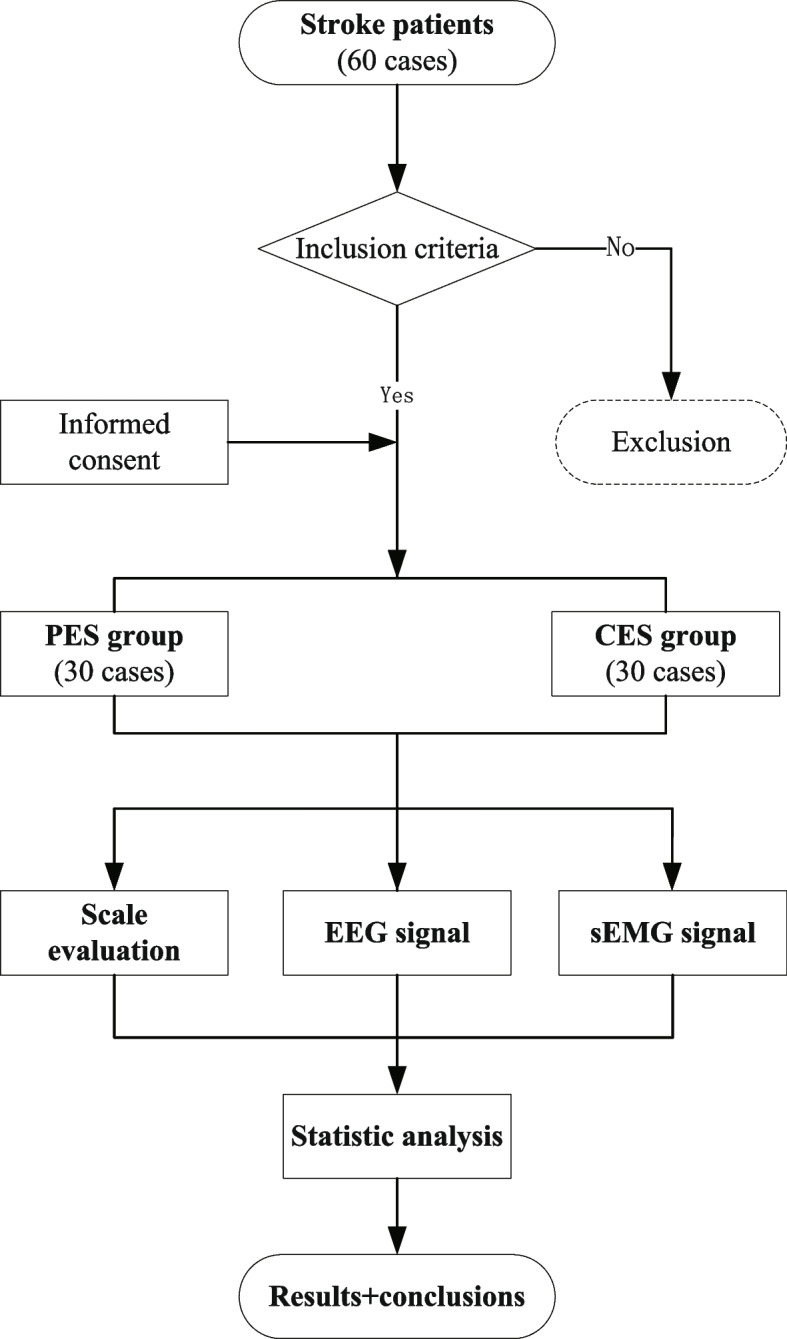
Flow diagram of the PES group and CES group. MBI, Modified Barthel Index; CSS, China Stroke Scale; U-FMA, upper Fugl-Meyer motor function assessment; MMT, Manual Muscle Testing; Brunnstrom, Brunnstrom motor function assessment

**Fig. 3:**
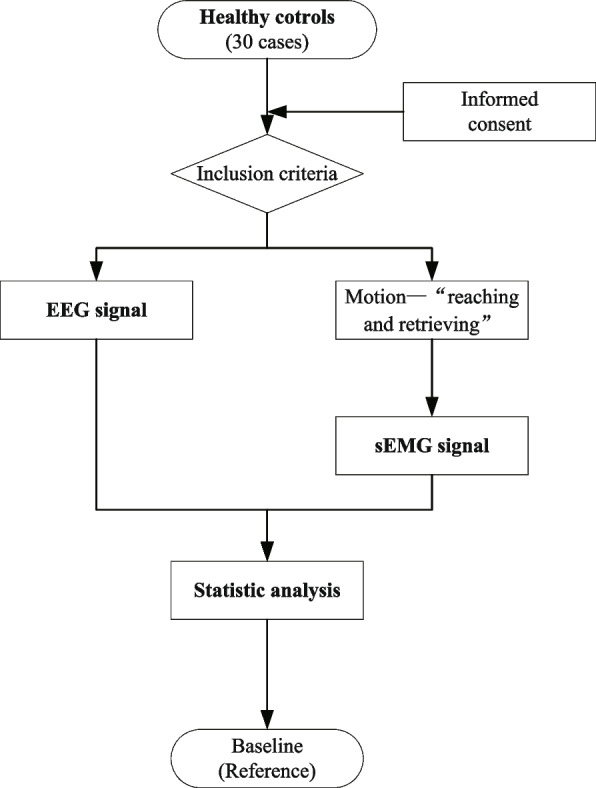
Flow diagram of the healthy control group

This clinical trial is designed as a double-blind RCT with 90 participants (60 stroke patients and 30 healthy controls) to be recruited. SPIRIT checklist and CONSORT statement were used when writing this study protocol [13, 14]. The whole study duration is about 17 weeks, including the washout period (7 days), the screening observation (3–7 days), the treatment period (3 weeks), and the follow-up period (3 months) (Fig. 1). After signing the informed consent (version date: 2021.11.19, version number: V1.1), all patients will be randomized divided into two groups in a blind manner—programmed flexor–extensor alternating electrical acupoint stimulation group (PES group) and conventional flexor–extensor alternating electrical acupoint stimulation group (CES group) with 30 cases in each, and the treatment will last for 3 weeks (6 days of treatment with 1-day rest per week). Healthy controls are used as a baseline without treatment. Participation in the trial is entirely voluntary, and they can withdraw at any time during the study without any reason. The participants are blinded, and the signal acquisition, evaluation, and outcome assessors are blinded. Because the treatment operation is too programmed, it is difficult to blind the performers. To prevent unblinding, subjects are advised not to discuss treatment options with evaluators (Figs. 2 and 3). All the patients will be followed up for 3 months. The follow-up time points will be at the end of the 1st month and 3rd month after the trial. The data and results will be monitored by the Data and Safety Monitoring Committee (DSMC) and the Ethics Committee.

### Study setting

The patients are stroke inpatients meeting the inclusion criteria in Acupuncture and Moxibustion Ward 3 of the Second Affiliated Hospital of Heilongjiang University of Chinese Medicine in Harbin, China. This hospital is the highest level one in China.

### Patients and healthy controls

All the 60 eligible patients will be recruited through clinic centers, hospital announcements, and China’s pubic social network platform (*WeChat* application), and the enrollment time is expected to be 3–5 months. After being informed of the possible problems during the participation in this trial, they should sign the informed consent and will be assessed by sEMG [15], resting state EEG with closed eyes [16], and scales. And the patients will be randomly assigned to either group. All patients will receive conventional medication, rehabilitation, and acupuncture. As informed, during the study period, any other Chinese and Western medicine or treatment care related to this disease will not be permitted without the approval from the trial staff in advance.

The healthy controls only need the detection of sEMG and EEG. Neither patients nor healthy controls will be involved in the design, conduct, or data processing of this study. The inclusion criteria and exclusion criteria of patients and healthy controls are presented in Table 1.

**Table 1:** The inclusion criteria and exclusion criteria

Inclusion criteria	Exclusion criteria
**Inclusion criteria for patients**	
a. Meet the diagnostic criteria of stroke [15, 16]	a. Severe cognitive dysfunction, severe aphasia, cannot cooperate with the whole treatment or testing process
b. Age: 35 ~ 75 years old, male and female	b. Patients with serious primary diseases such as heart, lung, kidney, liver, and endocrine system
c. Course of disease: 2 weeks to 3 months after stroke, stable vital signs, basically normal cognitive function, can cooperate to complete the test	c. Neurological or musculoskeletal diseases affecting functional recovery before onset
d. Manual muscle test (MMT) ≥ 2 [17], modified Ashworth (MAS) [18] of paralyzed upper limbs are graded I ~ II	d. Cerebral stem stroke or bilateral stroke
e. Body mass index (BMI) ≤ 28	e. Patients with severe anxiety, depression, affective disorders, schizophrenia, and other serious mental disorders
f. No serious heart, lung, kidney, and other functional damage; serious underlying diseases; pain in the affected side of the upper limb joint	f. Examination confirmed by brain tumor, brain trauma, brain parasitic diseases, metabolic disorders, rheumatic heart disease, coronary heart disease, and other heart diseases with atrial fibrillation caused by cerebral embolism
g. The informed consent is signed by the patient and/or his/her family members. Note: patients who meet the above 7 criteria can be included in this study	g. Patients with skin damage, infection, or deformity at the treatment site
**Inclusion criteria for healthy subjects**	
a. Those who have been proven to be healthy by physical examination and have no organic lesions or obvious functional diseases	
b. Age: 35 ~ 75 years old, male and female	
c. No cold, fever, cough, headache, and other physical abnormalities during the test	
d. Do not take any excitability drugs in the past 1 month, no recent treatment related to the trial content	
e. No history of mental or nervous system	
f. The subject agrees and signs the informed consent	

### Interventions

All patients received conventional medication, rehabilitation, and acupuncture. As informed, during the study period, any other Chinese and Western medicine or treatment care related to this disease will not be permitted without the approval from the trial staff in advance.

### PES group

**Fig. 4:**
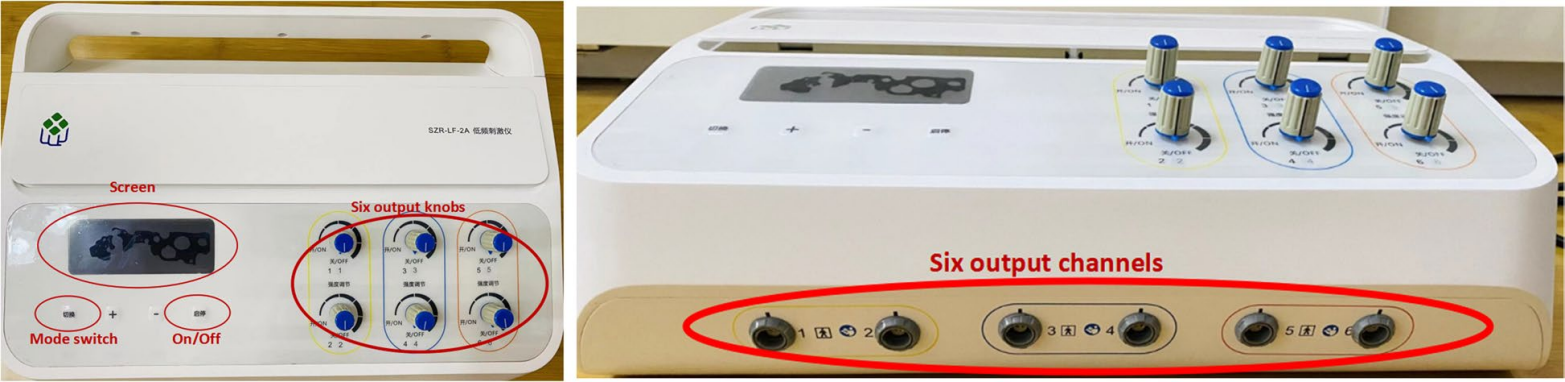
The fourth generation of the SZR-LF-2A low-frequency acupoint electric stimulation therapy instrument (patent no. ZL201610793646.9)

**Fig. 5:**
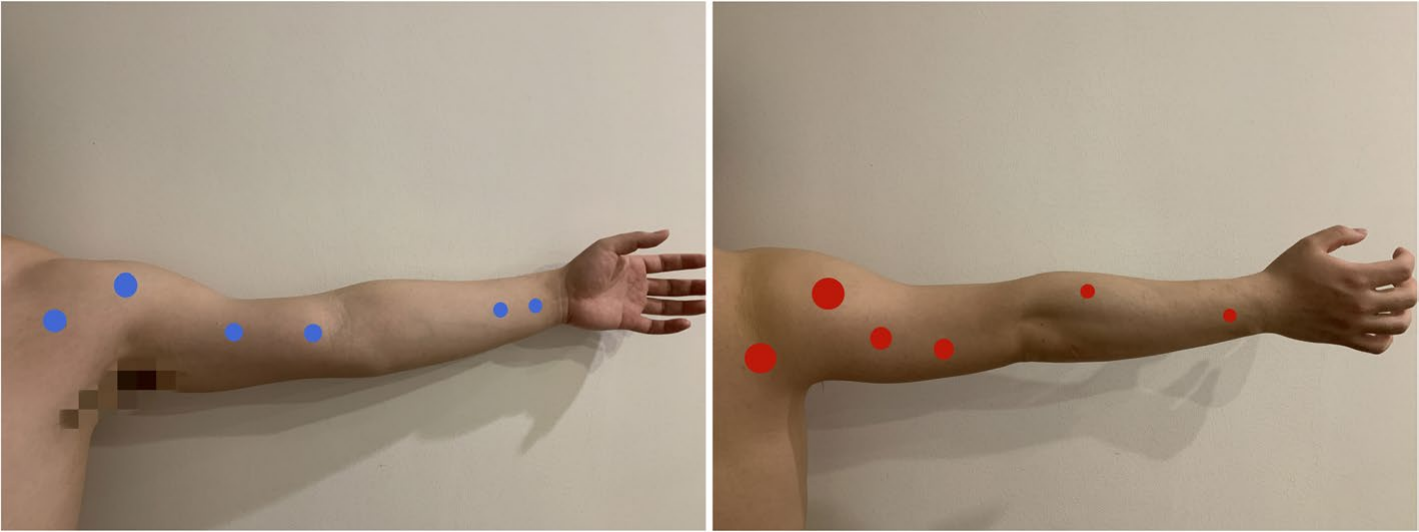
Position of the flexor electrodes and extensor electrodes (photos are provided by one author of this article)

Based on the routine treatment in the department of acupuncture [17], the fourth generation of SZR-LF-2A low-frequency acupoint electric stimulation therapy instrument (patent No. ZL201610793646.9) jointly developed by our research group and the Robotics Institute of Harbin Institute of Technology will be used to improve the program (Fig. 4). The output of the improved instrument program is the patient should complete the motion of “reaching and retrieving,” namely, shoulder joint forward flexion—elbow extension—wrist dorsal extension—finger extension (“reaching for objects”), then grasping—wrist flexion—elbow flexion—shoulder joint backward extension (“retrieve objects”). All patients are in a sitting position, the wrist of the affected limb is suspended, and the other parts do not touch any plane. The electrode connection is divided into six groups, with two patches in each group, and acupoints on the affected side are selected. Power on after the connection and the current is within the patient’s tolerance, no pain. Acupoint selection is as follows (Table 2, Fig. 5).

**Table 2:** Acupoint selection

Joints	Flexor acupionts	Extensor acupionts
Shoulder	Jianqian^a^, Zhourong (SP 20)	Naoshu (SI 10), Tainzong (SI 11)
Elbow	Tianquan (PC 2), Quzhou point^b^	Naohui (SJ 13), Xiaoluo (SJ 12)
Elbow	Ximen (PC 4), Neiguan (PC 6)	Shousanli (LI 10), Waiguan (SJ 5)

^a^Jianqian: an extra point of the upper extremities

^b^Quzhou point (0.5 in. outside and 2 in. above Quze [PC 3], between the long and short heads of brachial biceps)

### CES group

Based on the conventional treatment in the Department of Acupuncture, the output mode of the fourth generation of low-frequency acupoint electric stimulation instrument of the original program will be adopted to synchronously stimulate the flexor and extensor acupoints respectively. The selection of acupoints, treatment time, and course will be the same as those of the PES group.

### Healthy control group

No treatment, as the baseline. Ask them to do the “reaching and retrieving” gross motion and record the resting state EEG and motion state sEMG parameters.

All the patients will receive free medical care during the study period, including counseling, therapy, electrophysiology, and scale evaluation. Participation in the trial is entirely voluntary, and patients can withdraw at any time during the study without any reason. The patient’s medical records will be kept in the hospital and only available to researchers, research authorities and ethics committee members of the Second Affiliated Hospital of Heilongjiang University of Chinese Medicine for review. Any public reporting of the results will not disclose the patients’ and healthy controls’ privacy. If the participant withdraws, their data will also be recorded but will not be used in this study.

Furthermore, for improving the adherence, the relevant staff will adequately explain the treatment method, duration, potential risks, and other details to the participants and keep connected whenever they need any medical service.

### Randomization and blinding

A random grouping method will be adopted. The SPSS 26.0 statistical software is used to randomly generate serial numbers and groups, and blind selection cards are made and put into envelopes (with no difference in appearance). Numbers are according to the inclusion sequence of the subjects, and corresponding experimental groups are entered according to the blind selection cards. Envelope administrators will not participate in this study, and the staff who generate the allocation sequence, enroll participants, and assign participants to interventions will be different ones without communication about the allocation. When patients are treated, the operators should follow the treatment assignment.

The study will be conducted in a double-blind method. Due to the particularity of the operation, the operators (participating doctors and physiotherapists) will not be blinded, but only the electrophysiological indicator collectors, scale evaluators, data recorders, statistical analysts, outcome assessors, and the included subjects are blinded. The operators are not involved in the design of the protocol; thus, they have no determination of the potential differences of each treatment method. They only need to ensure the protocols are followed accurately, so that the treatment and results will not be influenced even though they are unblinded.

### Measurement

*Patient demographics*: age, gender, BMI, course of disease, functional damage and serious underlying diseases, and date of admission.

*Healthy control demographics*: age, gender, BMI, once eyes-closed resting state EEG signals, and real-time sEMG signals.

*Treatment information*: enrollment time, treatment time, hospital discharge time, withdrawal time, problems in the treatment period, scale evaluation (MBI, CSS, FMA-U, MMT, and Brunnstrom), eyes-closed resting state EEG signals, and real-time sEMG signals [19].

*Follow-up*: dates of follow-up visit, adjuvant therapies, overall health status at and during follow-up (recovery condition of the upper limb, activity of daily living, fundamental diseases, drugs, death, loss of follow-up), and scale evaluation (MBI, CSS, FMA-U, MMT, and Brunnstrom). The follow-up staff will also be blinded.

The trial time enrollment schedule is estimated to be 6–8 months with a 3-month follow-up after the end of treatment. In 48 h after signing the informed consent, the patients will be randomly divided into groups according to the order in the clinic and then receive treatment after being evaluated (Table 3).

**Table 3:** Schedule of study procedures

**Study period**	**Washout**	**Observation**	**Baseline**	**Treatment**			**Follow-up**	
Timepoint	≦7 days	3-7 days	0 day	1 day*	3 week◈	3 week^⊙^	1 month	3 month
**Patients**								
Informed consent	×							
Eligibility screening			×					
Patients’essential information			×					
Allocation			×					
Primary outcome								
sEMG			×	×	×	×		
EEG			×	×	×	×		
sEMG synergy-coherence analysis			×	×	×	×		
EEG power spectrum analysis			×	×	×	×		
Secondary outcomes								
MBI			×			×	×	×
CSS			×			×	×	×
FMA-U			×			×	×	×
MMT			×			×	×	×
Brunnstrom			×			×	×	×
**Healthy controls**								
Informed consent			×					
Eligibility screening			×					
Patients’ essential information			×					
Primary outcome								
sEMG			×					
EEG			×					
sEMG synergy-coherence analysis			×					
EEG power spectrum analysis			×					
Blinding assessment				×	×	×		
Adjuvant therapy				×	×	×	×	×
Adverse events				×	×	×	×	×

### Outcome measures

#### Primary outcome measures

The primary outcomes are real-time sEMG signals [20] and eyes-closed resting state EEG signals [21].

##### Patients

Before and after the first and last treatment, the immediate alpha, beta, delta, and theta bands of the eyes-closed resting state EEG of patients in the two groups and ask the patients to do the “reaching and retrieving” gross motion, recording the real-time sEMG data of RMS (root mean square) and MF (median frequency). Data of the following are collected: biceps brachii (BB), triceps brachii (TB), extensor carpi radialis (ECR), brachioradialis (B), extensor digitorum (ED), and flexor carpi ulnaris (FCU) during the whole process of the motion. Computer software calculates iEMG (integrated electromyogram) and MPF (mean power frequency) values.

##### Healthy controls

Before the beginning of the whole clinical trial, the immediate alpha, beta, delta, and theta bands of the eyes-closed resting state EEG of the healthy controls in a quiet, without any interference laboratory will be recorded and ask them to do the “reaching and retrieving” gross movement, recording the real-time sEMG parameters (RMS and MF) of BB, TB, ECR, B, ED, and FCU during the whole process of the motion. The computer software calculates iEMG and MPF values.

#### Secondary outcome measures

Before and after the whole trial, the secondary outcome variables including MBI (a daily functional activity assessment) [22], CSS (a neurological deficits assessment) [23], FMA-U (a motor function assessment for upper limb) [24], MMT (a muscle strength assessment), and Brunnstrom (a limb function recovery assessment) [25] are evaluated.

##### Sample size estimation

Sample size estimation was carried out according to the study objectives and protocol, and the difference between RMS values of sEMG of BB was obtained from the pretest of group 1 and group 2 after treatment and the healthy group. The difference between the healthy group and group 1 was 167.61 and 68.17 for group 2, and the standard deviations were respectively 127.22 and 148.46. The study effect size is 0.8, *α* = 0.05, test efficiency 1 − *β* = 0.80, and the number of sample groups is two. The sample size of each group is 25, and the total sample size is 50. Considering the possibility of sample loss in the trial, the sample size should be increased by 20%. Finally, the total sample size of this study should be 60 and divided into two groups with 30 sample size for each group.

##### Management and safety monitoring

Before the initiation of this trial, the feasibility and innovation of this protocol have been discussed comprehensively in the seminar composed of five authoritative experts in the field. Then, the task will be divided, and the relevant personnel should receive training. To prevent treatment bias, all the staff including doctors, physiotherapists, group allocation workers, data collectors, data registers, and data processing analysts need to be trained in advance on the tasks they are responsible for. Participants are all anonymous in this trial, and their demographic information will be stored in a Word file with a password kept by the manager, and their trial data will be recorded in another Word file by relevant staff without any participants’ information. If there is any special circumstance, all trial personnel should be informed in advance, and approval should be obtained before checking the participants’ relevant records. The project manager, researchers, and experts from the Ethics Committee of the Second Affiliated Hospital of Heilongjiang University of Chinese Medicine are mainly responsible for the quality control of these trials.

In order to achieve the ability of standard operation procedure (SOP), filling in of case report form (CRF), Electronic Data Capture (EDC) system, and data entry. All data will be accurately double-entered into the EDC system, and the electronic CRF will be identical to the paper CRF. Moreover, multiple imputation will be used to deal with the missing data as part of the sensitivity analysis.

##### Trial conduct auditing

In order to timely discover and solve the problems encountered in the study duration, the Project Management Group will check the trial records and hold a group meeting to review trial conduct twice a week. Meanwhile, the Trial Steering Group (including acupuncture and rehabilitation physicians, instrument engineer, and data analysts) and Ethics Committee meet each week to monitor the progress throughout the whole trial period, which will be independent from the investigators and the sponsor.

##### Data statistical analysis

The SPSS 26.0 statistical software will be used for analysis by three-party statisticians. Data presentation: each measurement is presented as mean ± standard deviation if it conforms to a normal distribution, or median and quartiles if it does not. Intra-group comparisons: if the measurement data in each group conforms to a normal distribution, paired *T* test will be used for comparison between each group before and after treatment; if all data does not conform to a normal distribution, paired sample Wilcoxon rank sum test is used. Inter-group comparison: If the measurement data of conforms to a normal distribution, one-way ANOVA will be performed, and the LSD method will be used for comparison between the groups (Welch’s test will be used to correct Dunnett’s T3 method when the variance is not equal); if the data does not all conform to a normal distribution, non-parametric test will be performed (Mann–Whitney *U* method is applied for comparison between two groups, and Kruskal–Wallis *H* method is for comparison between multiple groups).

##### Responsibilities of each trial groups

Under the guidance of the study director, the trial design group extensively reviews references and information about the subject, designs and adjusts the trial protocol, and collates the clinical data. Detailed information from ward feedback is collected daily, summarized and recorded, and reported to the director twice a week if there are no exceptional circumstances, but cannot be involved in treatment, data collection, or assessment. 

The expert group provides comprehensive guidance on trial design, implementation, and analysis of the results, and the design group reports weekly to it on the progress if no special circumstances occur.

The Ethics Committee and DSMC are responsible for the approval of the trial design and the occasional monitoring of the trial.

Acupuncture and Moxibustion Ward 3 is the main site for the implementation of the trial, from which the patients are sourced and treated, with the medical and nursing staff and the rehabilitation therapists providing accurate operation. They undertake patient treatment, observation, assessment, EEG-sEMG data collection, recording, and dealing with emergencies, which will be summarized by the ward manager and feed back to the design team on a daily basis. If difficulties are encountered, advice is sought from the trial director or even submitted to the expert group for joint discussion.

The third party data processing team processes and analyses the data according to the objective data provided by the design group and is not allowed to ask about anything related to the trial due to blind consideration.

## Discussion

In the process of motor function recovery in stroke patients, flexor and extensor coordination disorder is a common symptom [26, 27]. At present, the theoretical basis of limb function rehabilitation of apoplexy mainly includes brain plasticity theory and limb movement control theory [28]. Patients show the spasticity pattern of the upper limb flexor and lower limb extensor [29]. Therefore, stimulation of the flexor or extensor muscle alone is not enough. Abnormal movement patterns can affect the recovery of motor function and cause wrong posture, which needs to be corrected timely.

Rehabilitation of the upper limbs is difficult and time-consuming. This instrument is combined with the advantages of FES and acupoints. By stimulating the corresponding acupoints of the active and passive muscle groups of the upper extremity at low frequency, the contractile ability of the muscles is enhanced, and the relaxation and contraction function of the active and antagonistic muscle groups is coordinated, to effectively improve the motor function of the affected limb and promote the recovery of the central nervous system.

Six months is a gold period for rehabilitation after stroke [30]. But due to the COVID-19 epidemic, treatment has affected many patients. Rehabilitation training should be initiated soon after the stroke condition stabilized for better recovery effect [31]. The instrument is portable, easy to use, non-invasive and painless, and low cost, which may be easy to be accepted at home or in the community.

This study is a double-blind RCT. The safety and efficacy of the instrument are beyond doubt after 20 years of clinical application and many clinical studies [32–34]. This study improved the previous electrical stimulation output strategy, formed into a programmed one. Through the evaluation of neuro-electrophysiology and scales, the curative effects will be visualized and objectified for better interpreting the difference in the two stimulation methods, which may provide a new reference for integrated traditional Chinese and Western medicine in the treatment of motor function recovery after stroke.

## Trial status

The recruitment began on 3 June 2022 and is expected to last for 2 months (version date: 2021.11.19, version number: V1.1).

## Data Availability

Trial data are available on reasonable non-commercial request to the investigator of the study.

## Abbreviations

RCT: Randomized controlled trial
FES: Functional electrical stimulation
TCM: Traditional Chinese medicine
PES: Programmed flexor–extensor alternating electrical acupoint stimulation
CES: Conventional flexor–extensor alternating electrical acupoint stimulation
EEG: Electroencephalography
sEMG: Surface Electromyography
MBI: Modified Barthel Index
CSS: China Stroke Scale
FMA-U: Fugl-Meyer Assessment Upper Limb
MMT: Manual Muscle Testing
GBD: Global Burden of Disease
ADL: Activities of Daily Living
SPIRIT: Standard Protocol Items: Recommendations for Interventional Trials
CONSORT: Consolidated Standards of Reporting Trials
DMSMC: Data and Safety Monitoring Committee
RMS: Root mean square
MF: Median frequency
BB: Biceps brachii
TB: Triceps brachii
ECR: Extensor carpi radialis
B: Brachioradialis
ED: Extensor digitorum
FCU: Flexor carpi ulnaris
iEMG: Integrated Electromyography
MPF: Mean power frequency
SOP: Standard operation procedure
CRF: Case Report Form
EDC: Electronic Data Capture
ANOVA: Analysis of variance
